# Slow progression of exudative age related macular degeneration associated with hypertrophy of the retinal pigment epithelium

**DOI:** 10.12688/f1000research.5683.1

**Published:** 2014-12-02

**Authors:** Jeffrey Stern, David Eveleth, Jennifer Masula, Sally Temple

**Affiliations:** 1 Neural Stem Cell Institute and Athghin Biotechnology Inc, Albany, NY, 12206, USA; 2 Capital Region Retina, PLlc, Albany, NY, 12206, USA

**Keywords:** macular degeneration, AMD, choroidal neovascularization, CNV, retinal pigment epithelium, RPE, AMD treatment interval, RPE wound healing

## Abstract

**Rationale:  **Choroidal neovascular (CNV) lesions in younger patients are often accompanied by the appearance of a surrounding ring of pigment that is associated with disease regression or slowed disease progression. In older patients with age-related macular degeneration (AMD), however, hypertrophy of the retinal pigment epithelium (RPE) is known to occur but has not previously been reported to be associated with CNV regression. This report describes the clinical course of a case series of AMD patients with pigment hypertrophy adjacent to CNV associated with stabilization of the CNV lesion.

**Methods:** A retrospective analysis of exudative AMD patients seen by a single retina specialist over a 7-year period.

**Results: **Retrospective analysis of 955 exudative AMD patients revealed pigment hypertrophy associated with CNV in 33 patients. A ring of pigment surrounded CNV in 6 of these. Three representative patients are presented to illustrate the decrease in macular edema, reduced fluorescein leakage and slowed CNV progression that was associated with a pigment ring around CNV in AMD. Pigment hypertrophy was associated with blocked fluorescein leakage and exudative AMD patients with a complete pigment ring maintained stable visual acuity, macular edema, fluorescein leakage and CNV lesion size without treatment for intervals of up to 21 months.

**Conclusion:  **We report slowed disease progression in AMD patients who develop pigment around CNV. The slow rate of disease progression in the AMD patient subgroup having a pigment ring is a factor to consider in determining the treatment interval for exudative AMD patients.

## Introduction

A rapid loss of vision in exudative age-related macular degeneration (AMD) occurs when choroidal neovascular membranes (CNV) grow into the overlying retinal pigment epithelium (RPE) and neurosensory retina. The natural course of CNV is generally continued growth until central vision is lost, with rare spontaneous resolution in exudative AMD ^1^. In contrast, young patients with CNV secondary to myopia ^2^, histoplasmosis ^3^, rubella ^4^ or other causes ^5, 6^ often undergo stabilization that is accompanied by pigment hypertrophy developing around the CNV lesion. Although pigment hypertrophy is well known to occur in AMD patients ^1, 7^, there have been no reports on pigment hypertrophy associated with CNV regression in AMD. Here we report 3 AMD patients who developed a ring of hyperpigmentation around CNV during treatment of the CNV lesion that was accompanied by CNV regression even after the treatment was withdrawn for periods of up to 21 months.

## Materials and methods

Pigment hypertrophy was noted during fundus photography and fluorescein angiography (FA) for exudative AMD patients seen by a single retina specialist over a 7 year period. From a total of 966 exudative AMD patients, 33 developed a ring of pigment around the CNV lesion. Written informed consent to show images for research purposes was obtained from these patients. A prominent ring of pigment in the absence of significant hemorrhage or fibrous proliferation was observed in 6 of the 33 patients, and 3 had an uninterrupted series of fundus, fluorescein angiography (FA) and optical computed tomography (OCT) images suitable for presentation. Fundus photographs and FA images were obtained with a Zeiss FF450 or Topcon 50X fundus camera and OCT images were obtained with Zeiss, Optos or Heidleberg devices. Treatments were standard clinical practice in the year that care was provided, which included thermal laser, visudyne photodynamic therapy (PDT) and anti-vascular endothelial growth factor (anti-VEGF) intravitreal injections of bevacizumab (Avastin), pegaptanib (Macugen) or ranibizumab (Lucentis). Treatment intervals were varied as described for each case.

## Description of cases

**Figure 1.**
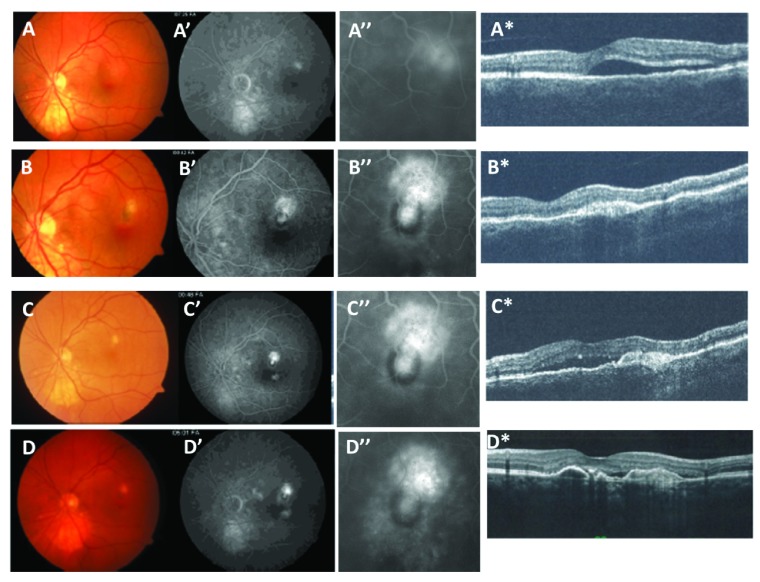
Partial pigment ring. Fundus photograph ( **A–D**), fluorescein angiogram (FA) mid-phase ( **A’–C’**), magnified FA ( **A”–D”**) and OCT ( A*–D*) images from a 58 year old female patient presenting with worsening visual acuity to 20/30 and metamorphopsia. Figures 1A-A*) Images taken prior to treatment show a peri-foveal fluorescein leakage with serous detachment due to CNV. The patient was treated with thermal laser to ablate the CNV lesion. Figures 1B-B*) After 6 months, CNV recurred in the supero-temporal aspect of the laser scar. The recurrence was treated with combined therapy of 2 PDT and 4 bevacizumab injections applied over a 6 month period. Figure 1C-C*) After remaining stable for 9 months without treatment, the CNV again recurred in the direction of the thermal laser scar but not in the direction of the partial pigment ring. This recurrence was treated with serial anti-VEGF antibody injections and then remained stable after 42 months with anti-VEGF therapy as shown in Figure 1D-D*.

The first case is a 58 year old woman who presented with metamorphopsia and worsening visual acuity to 20/30. The fundus photograph in Figure 1A, mid-phase FA in Figure 1A’, magnified FA in Figure 1A”; and OCT image in Figure 1A* were taken prior to treatment. These indicate perifoveal CNV with fluorescein leakage and serous detachment. The CNV was initially treated with focal thermal laser and the patient remained stable for 6 months, after which CNV recurred toward the fovea at the supero-temporal edge of the laser scar. Combined therapy with 4 bevacizumab injections (1.25mg/0.05ml) and 2 full dose visudyne PDT treatments were delivered over a 6-month period during which a pigment ring formed to partially surround the CNV lesion ( Figures 1B-B*). The patient then remained stable for 9 months without treatment. Following this period, serous fluid accumulated under the fovea with CNV recurring toward the original thermal laser scar in a direction away from the pigment ring ( Figures 1C-C*). This second CNV recurrence was treated with bevacizumab (1.25mg/0.05ml) or ranibizumab (0.5mg/0.05ml) every 4–6 weeks for 9 months during which time fluorescein leakage remained blocked in the direction of the pigment ring and active in the direction of the original thermal laser scar. The horizontal OCT images through the CNV lesion ( Figures 1C*-D*) indicate that serous fluid is replaced by RPE layer thickening. After 42 months of anti-VEGF therapy, fluorescein leakage remained contained in the direction toward the pigment ring with active leakage in the direction away from pigment.

**Figure 2.**
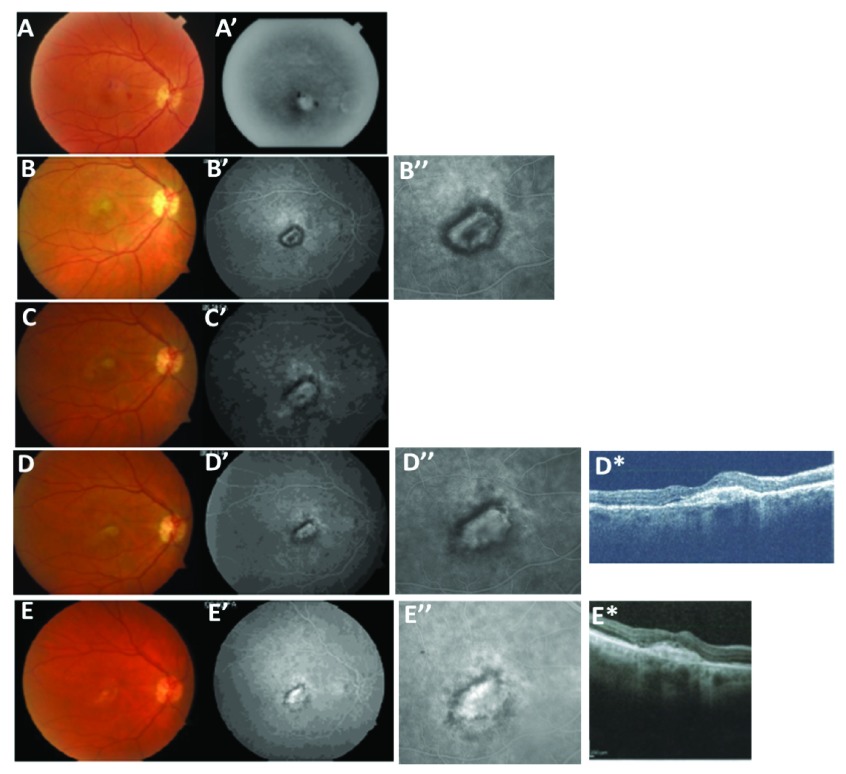
Recurrent pigment capping. ( Figures 1A-A’) shows a 71 year old female patient who presented with decreasing vision over 1 week with fundus photography and angiography indicating perifoveal CNV and a small amount of hemorrhage. Figures 2B-B”) After 5 visudyne PDT treatments over a 13 month period, a complete ring of pigment formed to surround the CNV and treatment was withheld. Note the reduced fluorescein leakage. Figures 2C-C’) The treatment interval was extended to 20 months after which symptoms and a small infero-temporal area of fluorescein leakage recurred. Anti-VEGF treatments with bevacizumab and pegaptanib were initiated and after 7 treatments over a 31 month period, a pigment ring re-formed around the CNV with elimination of the infero-temporal leakage as shown in Figures 2D-D*. After this, the treatment interval was again extended to 21 months without treatment during which the lesion remained stable as shown in Figures 2E-E*.

The second patient is a 71 year old woman who presented with metamorphopsia and decreased acuity to 20/50. Initial fundus photography and FA indicated a perifoveal classic CNV ( Figures 2A-A”). The CNV was treated with 4 full standard visudyne PDT sessions over a 13 month period. During this time, a ring of pigment formed to completely surround the CNV ( Figures 2B-B”). The patient then remained stable for 20 months without treatment after which symptoms and fluorescein leakage recurred ( Figures 2C-C”). Of note, the minimal leakage recurred where the pigment ring remained intact compared to more extensive fluorescein leakage where CNV broke through the infero-nasal aspect of the pigment ring. The patient then received 31 months of anti-VEGF therapy which resulted in the reformation of a complete pigment ring and deceased leakage ( Figures 2D-D*). The CNV lesion remained stable with minimal leakage and visual acuity stable at 20/60 for 21 months without treatment ( Figures 2E-E*).

**Figure 3.**
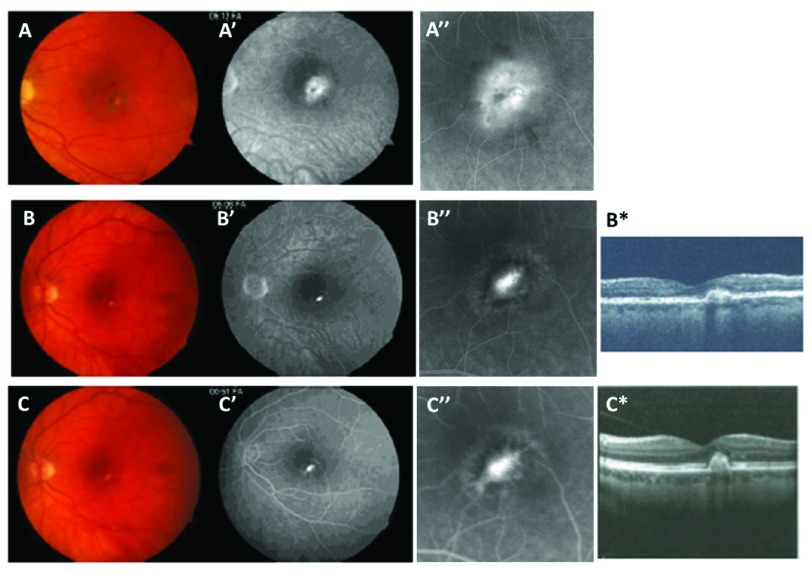
Stable pigment capping. ( Figures 3A-A”) show the initial fundus photographs and FA taken from a 48 year old woman presenting with metamorphopsia decreased visual acuity and a small, subfoveal CNV. After 5 anti-VEGF treatments with bevacizumab over a 9 month period, a pigment ring formed containing the leakage and treatment was withheld for 10 months until a small recurrence occurred toward the fovea as shown in Figures 3B-B*. Anti-VEGF treatments with bevacizumab were re-initiated and after 7 treatments over a 31 month period, a pigment ring re-formed around the CNV with elimination of the infero-temporal leakage. This was followed by a 10 month interval without treatment, during which the CNV again remained quiescent with a surrounding pigment ring as shown in Figures 3C-C*.

The third patient is a 48 year old woman who presented with metamorphopsia and vision loss to 20/40. The initial findings indicated CNV adjacent to the fovea ( Figures 3A-A”) for which anti-VEGF therapy with bevacizumab was initiated. After 5 injections over a 38 week period, leakage diminished and a dense pigment ring formed around the lesion ( Figures 3B-3B*’). Treatment was withheld and the patient remained stable for 10 months, but then complained of increasing metamorphopsia and was found to have recurrent CNV. The treatment was re-initiated for a 31 month period after which the pigment ring reformed and the treatment interval was again extended. The patient then remained stable with complete pigment capping, acuity of 20/20, and minimal FA leakage for 10 months without treatment ( Figures 3C-C*) after which she was lost to follow-up.

## Discussion

In this case series, 3 AMD patients developed a ring of pigment around CNV which was accompanied by decreased fluorescein leakage and slowed CNV growth in the direction of the pigment. A complete pigment ring was associated with much less rapid disease progression than expected ^1^, and patients with a complete ring were stable without treatment for extended periods. The presence of a pigment ring is known to be associated with CNV regression in younger patients ^2– 4^. Our case series suggests that the presence of pigment hypertrophy surrounding CNV can also be associated with slowed growth or regression of CNV in patients with AMD. We suggest that the presence of a pigment ring be considered in determining the treatment interval offered to AMD patients.

Wound repair of central nervous system tissues such as the RPE and neural retina is generally limited, yet evidence for RPE proliferation and wound repair has been described in young patients with CNV ^2– 6^, after RPE rips ^8, 9^, in animal models of laser-induced CNV ^10^, after RPE debridement ^11^, and to repopulate areas of RPE loss *in vitro* ^12^. It is possible that a proliferative RPE response to CNV has only recently become evident in AMD patients due to a prior lack of treatment to slow CNV growth that otherwise overwhelms the RPE response. The advent of new therapies to slow CNV growth may have altered the balance between CNV and RPE to unmask RPE wound healing. In both younger and older patients, increased pigmentation and thickening of the RPE layer is consistent with the hypothesis that CNV elicits a proliferative response in the RPE layer that strengthens the barrier against further CNV invasion. This RPE layer self-repair may be mediated by activation of a subpopulation of RPE stem cells that has been recently identified ^12^.

## Consent

Written informed consent to publish clinical images has been obtained from each patient.
