# Supplementary material for: Slow progression of exudative age related macular degeneration associated with hypertrophy of the retinal pigment epithelium
Source: F1000Res. 2014 Dec 2;3:293. [Version 1] doi: 10.12688/f1000research.5683.1 (PMC4314661; doi:10.12688/f1000research.5683.1)
Supplement: Peer review history

# Peer review: Referee response for version 1

**DOI:** 10.5256/f1000research.6075.r7254

**Published:** 2015-02-03

**Authors:** Igor Nasonkin

**Affiliations:** 1 Department of Ophthalmology, University of Pittsburgh, Pittsburgh, PA, USA

The article by Dr. Stern and colleagues is focused on summarizing cases of wet AMD with CMV lesions where Retinal Pigment Epithelium (RPE) undergoes self-repair associated with hypertrophy, slower disease progression and a ring of pigment forming around the CNV, caused by RPE proliferation.

This paper should be of interest to both clinicians and basic researchers working in regenerative ophthalmology. Age Related Macular Degeneration is the leading cause of vision loss in the world, and wet AMD pathology is very destructive to the fine layer of neural retina and RPE. While anti-VEGF antibody (bevacizumab) intravitreal injections is a powereful treatment to slow down vision loss caused by wet AMD, there is no regeneration of RPE at the site of initial CMV lesion and RPE tissue in this part of retina is considered to be lost forever. Cell transplantation (such as RPE derived form adult or embryonic stem cells) is one innovative way to approach RPE repair and treating vision loss in wet AMD. However, cell transplantation is a very invasive procedure per se and may precipitate further destruction of the fine and very sensitive layer of neural tissue such as neural retina/RPE. Finding innovative ways to induce the regeneration of RPE in patients with AMD will likely be a significant leap forward in treating this devastating disease.

Dr. Stern's 7 year old summary of observing patients with wet AMD and CNV identified a very interesting group of about 3.5 % of patients (33 out of 955 total), where spontaneous RPE proliferation took place, associated with a distinct ring of pigment around the CMV, decreased macular edema and stabilized vision. This indicates that some small percentage of patients are able to partially regenerate RPE at the site of lesion after initial RPE damage took place,  and provides a unique opportunity to identify the factors (secreted by CMV lesion, as well as genetic factors) enabling this selected cohort of patients  to initiate RPE regeneration (hypertrophy).

Though the response leading to the formation of so called pigment ring, does not lead to substantial regeneration of RPE, this nevertheless leads to stabilization of vision and does slow down the disease progression.  The report gives a unique tool to regenerative ophthalmology, as well as basic researchers focused on RPE biology, pathology and regeneration to identify such factors and pathways, which are activated in RPE in this cohort of patients, and enable RPE to reenter cell division and replenish and repair the RPE layer damaged by AMD. The next logical step seems to be finding the small molecules, which may mimic CMV signaling and initiate RPE proliferation in situ.  Dr. Stern's report provides unique tools to scientists working on AMD and retinal regeneration to find the innovative approaches to restore retina by stimulating the endogenous regeneration in adult RPE or/and in subpopulation of cells within RPE layer, which possesses stem cell signature and capable to better respond to proliferative signals from CMV lesion.

Collectively, this report is of substantial value and significance and should be indexed to enable researchers to find the mechanisms, controlling the endogenous regeneration of RPE.

I have read this submission. I believe that I have an appropriate level of expertise to confirm that it is of an acceptable scientific standard.

# Peer review: Referee response for version 1

**DOI:** 10.5256/f1000research.6075.r7253

**Published:** 2015-01-26

**Authors:** Thomas Gardner

**Affiliations:** 1 Kellog Eye Center, University of Michigan, Ann Arbor, MI, USA

The authors have extended prior clinical observations of RPE rings associated with resolution of sub retinal neovascularization in young patients and now find 3 unusual cases of similar findings in patients with AMD. These findings represent astute clinical observations and the combination with possible cellular pathophysiology and potential implications for treating AMD make this a worthy manuscript for consideration. The title and abstract are appropriate, the writing is clear and succinct, and the observations will be of use in the field. In my own observations I concur that the presence of RPE rings is a likely correlate of CNV regression.

I have read this submission. I believe that I have an appropriate level of expertise to confirm that it is of an acceptable scientific standard.
